# The effect of hematopoietic stem cell transplantation on patient-reported subjective oral dryness: a systematic review focusing on prevalence, severity and distress

**DOI:** 10.1007/s00520-023-07921-1

**Published:** 2023-07-08

**Authors:** Marjolein S. Bulthuis, Lucky L. A. van Gennip, Ewald M. Bronkhorst, Nicole M. A. Blijlevens, Marie-Charlotte D. N. J. M. Huysmans, Stephanie J. M. van Leeuwen, Renske Z. Thomas

**Affiliations:** 1 Department of Dentistry, Radboud University Medical Center, Nijmegen, The Netherlands; 2 Department of Hematology, Radboud University Medical Center, Nijmegen, The Netherlands

**Keywords:** Hematopoietic stem cell transplantation, Xerostomia, Subjective mouth dryness, Patient-reported oral dryness, Systematic review

## Abstract

**Objective:**

The aim of the present systematic review is to assess the prevalence and severity of and distress caused by xerostomia over time in adult hematopoietic stem cell transplantation (HSCT) recipients.

**Methods:**

PubMed, Embase, and the Cochrane Library were searched for papers published between January 2000 and May 2022. Clinical studies were included if patient-reported subjective oral dryness was reported in adult autologous or allogeneic HSCT recipients. Risk of bias was assessed according to a quality grading strategy published by the oral care study group of the MASCC/ISOO, resulting in a score between 0 (highest risk of bias) and 10 (lowest risk of bias). Separate analysis focused on autologous HSCT recipients, allogeneic HSCT recipients receiving a myeloablative conditioning (MAC), and those receiving a reduced intensity conditioning (RIC).

**Results:**

Searches yielded 1792 unique records; 22 studies met the inclusion criteria. The quality scores ranged between 1 and 7, with a median score of 4. The prevalence, severity, and distress of xerostomia increased shortly after HSCT. Severity of xerostomia in allogeneic MAC recipients was higher compared to allogeneic RIC recipients 2–5 months post-HSCT (mean difference: 18 points on 0–100 scale, 95% CI: 9–27); after 1–2 years, there was no significant difference anymore.

**Conclusion:**

The prevalence of xerostomia in HSCT recipients is high in comparison to the general population. The severity of complaints is raised during the first year post-HSCT. The intensity of the conditioning plays a key role in the short-term development of xerostomia, while factors affecting the recovery in the long term remain largely unknown.

**Supplementary information:**

The online version contains supplementary material available at 10.1007/s00520-023-07921-1.

## Introduction

Patient-reported subjective oral dryness, or xerostomia, is a common oral side effect from cancer therapies, especially from radiation therapy in the head and neck region [1]. Even though less extensively investigated, the prevalence of xerostomia might also increase after hematopoietic stem cell transplantation (HSCT) preceded by an intensive conditioning regimen. HSCT recipients reported higher levels of xerostomia compared to their partners [2]. Jensen et al. conducted a systematic review in 2010 on behalf of the Multinational Association of Supportive Care in Cancer (MASCC) and the International Society of Oral Oncology (ISOO). In this review, only three studies assessing xerostomia after HSCT could be included. A prevalence of 40% during treatment and 79% 7 years after treatment was reported [1].

Xerostomia is associated with several symptoms, like discomfort [3] and difficulty with speech and food intake [4]. Furthermore, xerostomia influences the quality of life negatively [1]; it is rated as one of the most bothersome symptoms [5, 6] and the main long-term oral complaint [7–9] by HSCT-recipients. Specific instruments are available to measure the severity of xerostomia, like the Xerostomia Inventory [10]. Furthermore, many questionnaires that measure quality of life or symptoms after cancer therapy include a question about mouth dryness, like some additional modules of the European Organization for Research and Treatment of Cancer (e.g. EORTC QLQ-OH15) [11].

Xerostomia is a patient-reported outcome that is inextricably linked with hyposalivation: an objectively reduced salivary flow rate. Some studies concluded that unstimulated flow rates were related to the subjective feeling of mouth dryness in HSCT recipients [12, 13], while other authors could not find a relation between reduced salivary secretion and subjective oral dryness [14]. Even though xerostomia is primarily caused by a decrease in the function of the salivary glands [4], other factors like oral mucosal moistness [15], which might be associated with mouth breathing [16] or saliva composition [17], contribute to the feeling of mouth dryness as well. Furthermore, it was suggested that neuropathic mechanisms are involved in the perception of mouth dryness [18]. The prevalence of xerostomia in HSCT recipients was up to 4.7 times higher than the prevalence of hyposalivation of unstimulated saliva [9, 12, 19] and 2.4 times higher than the prevalence of hyposalivation of stimulated saliva [12].

Salivary flow rates are decreased several days and months after HSCT, but appear to improve again over time [20]. As a result, it might be expected that xerostomia in HSCT recipients is transient in nature. This is in contrast to the prevalence of xerostomia after radiation therapy in head and neck cancer that shows an unchanged pattern between 1 month and more than 2 years after treatment [1].

Xerostomia has a multifactorial etiology and, in this specific population, might be the result of the acute toxicity of the conditioning regimen. Total body irradiation (TBI) as part of the conditioning regimen might have an additional effect compared to chemotherapy alone; it was suggested that TBI caused complaints during or immediately after its administration [21, 22] and that it was related to lack of recovery in the long term [23, 24]. Furthermore, the use of (xerogenic) medication [25], perceived stress [26, 27], or the development of chronic graft versus host disease (cGvHD) in allogeneic HSCT recipients, might also be related to long-term xerostomia [28].

The prevalence and severity of xerostomia after HSCT have not been systematically reviewed since 2010. Furthermore, it remains unclear whether patients that receive higher intensity conditioning regimens are at higher risk of developing xerostomia or whether complaints differ after autologous and allogeneic transplants. Therefore, the aim of the present systematic review is to assess the prevalence and severity of and distress caused by xerostomia over time, in adult HSCT recipients, and to determine whether type of transplantation and conditioning regimen influence the severity of xerostomia.

## Materials and methods

This systematic review was registered in PROSPERO (CRD42020168364) and followed the guidelines provided in the Transparent Reporting of Systematic Reviews and Meta-analysis (PRISMA) statement [29].

### Search strategy

The following databases were searched from January 2000 up to June 2021: the Cochrane Central Register of Controlled Trials (CENTRAL), MEDLINE via PubMed, and EMBASE via OVID. The search was updated in May 2022. A detailed and broad search strategy was designed with the help of a medical librarian. Because a preliminary search revealed that relevant papers were missed if xerostomia and oral or mouth dryness were used as only outcome terms, a combination of other terms (like: quality of life, side effects, and symptoms) was added to the search strategy (Table S1). In composite scales that assessed multiple systems, the relevant question on xerostomia was used. The reference lists of included studies were examined to identify additional studies. Duplicate references were identified and removed with the help of EndNote X9 software.

Cohort studies, controlled trials, and cross-sectional studies were considered for inclusion if they reported on xerostomia, defined as patient-reported subjective oral dryness, in a population of adult HSCT recipients. Studies were only included if at least 80% of the subjects reached adulthood during HSCT and all subjects were ≥ 18 years at the examination. The time since HSCT should be clearly specified, comprising the following: studies reporting on xerostomia in the first year post-HSCT were only included if the outcome was reported within a range of 1 month. An exception was made when xerostomia was reported at a specific phase of treatment (e.g., discharge from the hospital). No specific time range was required for studies reporting xerostomia > 1 year post-treatment. Prevalence, mean severity, and mean distress scores were the outcomes of interest. Studies were excluded if patients were selected based on complications that developed after HSCT, like cGvHD or poor general health. Furthermore, studies were excluded if authors only reported on objective oral dryness, oral dryness based on clinical characteristics as diagnosed by a doctor or dentist, or combined with taste alterations or food intake (e.g. toxicity criteria of the Radiation Therapy Oncology Group [30] or the Common Terminology Criteria for Adverse Events [31]).

### Screening and selection

Two review authors (MB and LvG) independently screened titles and abstracts for eligibility. Full-text copies of potentially relevant publications were obtained, even in case of disagreement or doubt. The full-text copies were screened for the words “xerostomia” and “dry.” If papers reported on a potentially eligible outcome, full-texts were read to establish whether they met the inclusion criteria.

### Data extraction and quality assessment

Data on study design, country, participants (age, diagnosis), treatment (type of HSCT, intensity of the conditioning regimen, and TBI), time since HSCT, measurement instrument (questionnaire and recall period), and outcome data (prevalence, severity, or distress scores) were extracted from the papers. If one of these variables was missing, the corresponding author was contacted in an attempt to obtain additional data. When outcomes were only graphically shown, data was extracted with a ruler from enlarged graphs, but only when authors were not reached or underlying data was no longer available. If applicable, authors were asked to divide populations of sufficient participants (score 1 or 2 for estimate precision, Table S2) into relevant subgroups.

Quality of the reported outcomes was assessed by two review authors (MB and RT) according to a quality grading strategy published by the oral care study group of the MASCC/ISOO [32]. The grading strategy, including adaptations made to fit the current research question, can be found in Table S2. Study characteristics that might have resulted in different forms of bias were numerically scored, which resulted is a score between 0 (highest risk of bias) and 10 (lowest risk of bias). The quality scores were categorized as follows:

-  ≤ 3 points: high risk of bias
- 4–6 points: moderate risk of bias
-  ≥ 7 points: low risk of bias

Only outcomes with a low or moderate risk of bias were included in the meta-analysis.

### Statistical analysis

Results over time are graphically shown for three outcomes separately: prevalence, mean severity, and mean distress. Prevalence was defined as the percentage of patients that experienced xerostomia of all extents, based on questions using a yes/no format or ordinal questionnaires calculating the proportion of patients that report at least “a little,” “mild,” or “slight” xerostomia. Severity, in some papers also called intensity, referred to the extent to which xerostomia was experienced by patients, measured on ordinal or continuous scales. Distress referred to the degree to which the patient was bothered by the symptom, also measured on an ordinal or continuous scale. In order to be able to combine results from different studies, severity and distress scores were recalculated to a scale from 0 to 100 using the following formula:

$$\mathrm{Rescaled}\;\mathrm{severity}/\mathrm{distress}\;\mathrm{scores}=\frac{\mathrm{reported}\;\mathrm{mean}\;\mathrm{score}-\mathrm{lowest}\;\mathrm{response}\;\mathrm{option}}{\mathrm{range}\;\mathrm{between}\;\mathrm{highest}\;\mathrm{and}\;\mathrm{lowest}\;\mathrm{response}\;\mathrm{option}}\ast100$$

In the “Results” section, only rescaled results are reported. The following time periods were chosen to report on xerostomia:

- Baseline: before the conditioning regimen and stem cell infusion
- Week 1: first week after HSCT, some papers refer to “neutropenia” or “nadir”
- Week 2–4: continuation of hospitalization phase, including discharge in some studies
- 1–2 months post-HSCT: usually the first measurement after discharge
- 2–5 months post-HSCT
- 5–8 months post-HSCT
- 1–2 years post-HSCT: long-term
-  > 2 years post-HSCT: long-term

### Data synthesis

Meta-analyses of rescaled severity scores were performed to facilitate the interpretation of data summarized in graphs. Forest plots were developed with Review Manager software (version 5.3) and summarized as mean differences (MD) and their 95% confidence intervals (95% CI). A fixed-effects model was only used if statistical heterogeneity was judged to be limited; otherwise, a random-effects model was chosen. Meta-analyses were conducted to answer two different research questions:

- To determine changes from baseline, within-study changes were calculated over different time intervals. These within-study changes were aggregated with the help of Review Manager, to summarize the overall change from baseline. Given the demanded experiment vs. control structure, an imaginary control group was entered to Review Manager, with a mean of 0, SD of 0.00001, and *N* of 1,000,000. If SDs from change scores were not reported, the SD was imputed as suggested in the Cochrane Handbook for systematic Reviews of Interventions, chapter 6 [ 33]. In summary, the SD of the change score was imputed based on the known SD from baseline and the follow-up measurement and a correlation coefficient calculated from another study reporting the SD of change scores (if more studies were available, the study with the highest quality was chosen to calculate the correlation coefficient). MDs were calculated between baseline and week 1, 2–5 months, and 1–2 years post-HSCT respectively. These time intervals were chosen taking into account the number and the quality of the available studies and the spread over time.
- To determine differences between subgroups, MDs within studies were calculated per time period. These MDs between subgroups within studies were aggregated. MDs were only calculated if at least 3 studies, comparing two subgroups of interest, could be included per time period.

### Risk indicators and subgroups

To determine the influence of type of transplantation and conditioning regimen, the following risk indicators were defined:

- Type of transplantation: autologous or allogeneic transplantations
- Intensity of the conditioning regimen: myeloablative (MAC) or non-myeloablative or reduced intensity (RIC)
- For allogeneic transplantations: the development of cGvHD
- Type of the conditioning regimen: chemotherapy or chemotherapy in combination with TBI

For the following subgroups, enough data was available to report data separately: autologous recipients, allogeneic recipients receiving MAC, and those receiving RIC. A narrative approach was used to summarize data on the influence of TBI and cGvHD, because only limited data was available on these risk indicators.

## Results

**Fig. 1:**
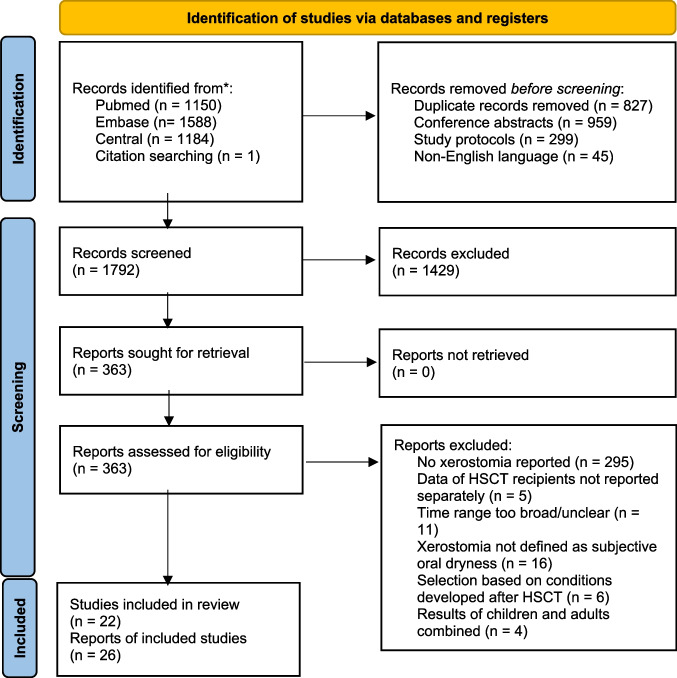
PRISMA flow diagram [29]

Electronic searches and citation searching retrieved 3923 references. After removing duplicates, conference abstracts, study protocols, and non-English papers, 1792 unique publications remained. Screening of titles and abstracts resulted in discarding of 1429 records. Full-text copies of the remaining 363 publications were obtained, and of these, 295 publications were excluded because no outcome of interest was reported. Full-text reading of the remaining 68 publications resulted in the exclusion of another 42 publications that did not meet the inclusion criteria (Table S3). Study characteristics of the 22 included studies (26 references) are listed in Table 1. The flow diagram of this process is presented in Fig. 1 [29].

**Table 1:** Characteristics of included studies

	Median age (range) at study	Diagnoses^1^	Treatment	Questionnaire (time frame)	Xerostomia outcome	Time points of xerostomia measurements
Abasaeed 2018 USA [34] Longitudinal	49	AML; MM; MDS; ALL; other	7 autologous 16 allogeneic 20 MAC; 3 RIC 39% TBI	QLQ-H&N35 (last week)	Severity	• Pre-transplant • Day 30 (± 5) • Day 80 (± 5)
Andersson 2008, 2009, 2011 Sweden [5, 23, 35] Longitudinal	54 (19–70)	Lymphoma; MM; AL; CL; solid tumors; other	145 autologous 57 allogeneic 167 MAC; 32 RIC 9% TBI	QLQ-HDC-19 (last week)	Prevalence Severity	• Inclusion • Baseline • 1 month post-HSCT • 3 months post-HSCT^2^ • 6 months post-HSCT^2^ • 12 months post-HSCT
Arduino 2022 Italy [19] Cross-sectional	Mean: 54 (SD: 11)	AL/MDS; MM; lymphoma/CLL; other	32 allogeneic 12 MAC; 20 RIC 47% TBI	Asking if xerostomia was present	Prevalence	> 2 years post-HSCT 49 months (SD 11) post-HSCT
Bennett 2015 USA [36] Longitudinal	Mean: 55 (SD: 14)	MM; AML; MS; CLL/PSS; ALL; NHL; other	18 autologous 14 allogeneic 25 MAC; 7 RIC	PRO-CTCAE (last week)	Severity^2^	Hospital: weeks 1, 2, 3, and 4 Post-hospital: weeks 1, 2, 3, and 4
Bergkvist 2015 Sweden [37] Cross-sectional	At HSCT: 44 (19–61) At study: 49 (21–65)	AL; CL; MPD/MDS lymphoma; myeloma; other	117 allogeneic 69 MAC; 48 RIC 42% TBI	SFID-SCT (last week)	Prevalence	Median 5 (1–11) years post-HSCT
Cheon 2021 South Korea [38] Cross-sectional	45 (21–70)	AML; ALL; AA; MS; other	67 allogeneic 17 MAC, 50 RIC No TBI	Is your mouth dry because your saliva has decreased?	Prevalence	> 2 years post-HSCT Median 25.7 months
Edman 2001 Sweden [6] Cross-sectional	Mean: 39 (22–62)	CML; AML; other	25 allogeneic 25 MAC^6^ 88% TBI	SFID-BMT (last month)	Prevalence	2–4 years post-HSCT
Eriksson 2022 Sweden [39] Longitudinal	52 (18–65)^3^	AL; MDS; CL; lymphoma, plasma cell disorder; other	195 allogeneic 61 MAC, 134 RIC 29% TBI	SFID-SCT (last week)	Prevalence Severity^3^ Distress^3^	• Baseline • 4 months post-HSCT • 7 months post-HSCT • 13 months post-HSCT
Ferreira 2020 Brazil [40] Longitudinal	53 (19–75)	NHL; MM; AML; CML; MS; ALL; Crohn’s disease; other	31 autologous 20 allogeneic 51 MAC^6^ 10% TBI	QLQ-H&N35 (last week)	Severity^3^	• Before conditioning • During neutropenia
Hayden 2004 Ireland [41] Cross-sectional	At HSCT: 36 (14–55) At study: adults	CML	46 allogeneic 46 MAC^3^ 29% TBI	QLQ-Leu (last week)	Prevalence Severity	Median 98 months (34–217) post-HSCT
Iestra 2002 The Netherlands [42] Longitudinal	Mean (SD) Completers: 44 (11) Dropouts: 40 (11)	Leukemia; MM; other	60 autologous 58 allogeneic 118 MAC^6^ 64% TBI	Self-designed questionnaire (> 2 days in last 14 days)	Prevalence	• Day 50 (all discharged) • Day 75 • Day 125 • Day 200 • Day 350
Jones 2013 USA [43] Longitudinal	Mean: 62 (SD 7)	MM	66 autologous 66 MAC no TBI	MDASI-MM (past 24 h)	Prevalence (moderate/severe)^2^ Severity	• Before conditioning • 7 days post-HSCT
Kirsch 2014 Switzerland [44] Cross-sectional	At HSCT: 43 (18–68) At study: 52 (20–76)	AML/CML; ALL/CLL; MS/MPS; HL/NHL; plasma cell disorder; other	361 allogeneic^4^ 272 MAC, 86 RIC 58% TBI	PROVIVO (last week)	Prevalence^3^ Severity^3^ Distress^3^	≥ 1 year post-HSCT Median 7.1 year (1–33)
Kolke 2019 USA [45] Longitudinal	Mean: 58 (SD 12)	Lymphoma; MM, leukemia; MS; other	33 autologous 21 allogeneic 40 MAC, 14 RIC^6^ 4% TBI	MSAS-SF (last week)	Prevalence^3^ Distress^3^	• Prior to hospitalization • Nadir, hospital days 7–13 • Discharge, hospital days 14–24
Larsen 2003, 2004 Sweden [46, 47] Longitudinal	45 (18–65)	Breast cancer; CL; AL; MM; other	26 autologous 17 allogeneic 43 MAC 42% TBI	SFID-SCT (moment of answering)	Prevalence Severity^2^ Distress^2^	• Admission, day − 11 to − 1 • Day before conditioning • Day of HSCT • Start protective care, days 1–6 • Mid protective care • End protective care, days 8–29 • Discharge, days 11–57
Lockhart 2005 USA [48] Longitudinal	Mean (SD) Pilocarpine: 48 (11) Placebo: 46 (10)	Breast cancer; lymphoma; MM; AML; other	36 autologous^5^ 36 MAC, 11% TBI	VAS (most of the time, asked every day)	Severity (mean and max)^2^	From day 1 of chemotherapy till day 10
Naegele 2018 Germany [49] Longitudinal	61 (43–74)	MM	29 autologous 29 MAC no TBI	PROVIVO (last week)	Prevalence Severity Distress	• Admission, day − 4 to − 3 • Nadir, days 5–8 • Discharge, days 14–21 • 30 days after discharge, days 43–55
Vellenga 2001, van Agthoven 2001 [50, 51] The Netherlands Longitudinal	ABMT: 50 (18–63) PSCT: 51 (18–64)	NHL; HL	118 autologous 118 MAC no TBI	RSCL (last week)	Distress	• 14 days post-HSCT • 3 months post-discharge
Warchala 2019 Poland [52] Longitudinal	Mean: 40 (SD 13)	AML; ALL	60 allogeneic	RSCL (last week)	Distress	Discharge (days from HSCT not reported)
Watson 2004 United Kingdom [53] Cross-sectional	Age at entry Autologous: 37 (15–52) Allogeneic: 32 (16–49) ≥ 18 at study	AML	74 autologous 97 allogeneic 171 MAC^6^ 81% TBI	QLQ-Leu (last month)	Prevalence	≥ 1 year post-HSCT Median 14 months (IQR 12–22)
Wood 2013 USA [54] Longitudinal	58	AML; MM; NHL; ALL; MDS; other	10 autologous 22 allogeneic 21 MAC, 11 RIC No TBI	PRO-CTCAE (last week)	Severity^3^	• Baseline (day − 20) • Day 0 • Day 7 • 2–4 weeks (mean, days 14–28)^3^ • 1–2 months (mean, days 35–56)^3^ • 2–5 months (mean, days 63–100)^3^
Wysocka-Słowik 2021 Poland [55] Longitudinal	Mean: 47 (19–69)	AML	80 allogeneic 54 MAC, 26 RIC	Authorial questionnaire	Prevalence Severity	• Day − 10 to day − 7 • Day + 3 to day + 7 • Day + 8 to day + 14

Abbreviations: *ABMT*, autologous bone marrow transplantation; *PSCT*, peripheral stem cell transplantation; *AA*, aplastic anemia; *AL*, acute leukemia; *ALL*, acute lymphoblastic leukemia; *AML*; acute myeloid leukemia; *CL*, chronic leukemia; *CLL*, chronic lymphocytic leukemia; *CML* chronic myeloid leukemia; *HL*, Hodgkin’s lymphoma; *MDS*, myelodysplastic syndrome; *MM*, multiple myeloma; *MPS*, myeloproliferative syndrome; *MS*, myelodysplastic syndrome; *NHL*, non-Hodgkin’s lymphoma; *PSS*, progressive systemic sclerosis; *MAC*, myeloablative conditioning; *RIC*, reduced intensity conditioning; *TBI*, total body irradiation; *HSCT*, hematopoietic stem cell transplantation; *IQR*, inter quartile range

^1^Diagnoses are ordered from high prevalence to low prevalence; diagnoses that count for ≤ 5% of the population are included in the category “other”

^2^Data extracted from graph (Andersson: only subgroups after 3 and 6 months)

^3^Additional data provided by the authors

^4^15 patients that were < 18 years old at HSCT were excluded from the dataset

^5^Only data of 16 patients that received no pilocarpine were included

^6^Doses of the conditioning regimen not reported, the distinction between MAC and RIC was estimated by a hematologist

Authors of 18 studies were contacted in an attempt to obtain additional information. We were unable to reach four authors [23, 43, 52, 55]; four authors replied that data was no longer available [37, 47, 48, 50]. Four authors provided additional information [19, 36, 38, 41], three authors provided additional data [40, 45, 49], and three authors provided individual patient data [39, 44, 54].

Two randomized clinical trials [48, 50], 13 longitudinal cohort studies, and seven cross-sectional studies were included. The majority of the studies was conducted in Europe, while six studies were conducted in the USA, one in Asia, and one in Brazil. The age of the included patients varied considerably between and within the included studies. Several studies reported a mean or median age of below 40 at HSCT [6, 41, 53], while others included patients with a median or mean age of above 60 [43, 49]. Patients were diagnosed with a variety of, mostly hematological, diseases, while some studies included patients with solid tumors as well.

### Quality of reported outcomes

Quality scores of outcomes as reported in the included papers are listed in Table S4. Quality scores ranged between 1 [19] and 7 [23], with a median score of 4. If applicable, subgroups were scored separately because smaller numbers of patients could lead to a lower estimated precision. One study reported outcomes with a low risk of bias [23], the majority was classified to have a moderate risk of bias, and seven were classified to have a high risk of bias [6, 19, 38, 41, 48, 52, 55].

### Prevalence of xerostomia

**Fig. 2:**
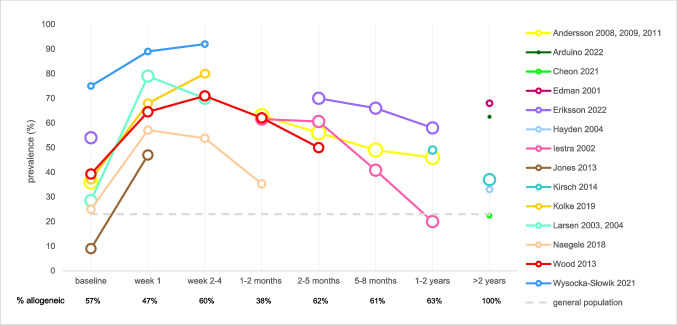
Prevalence of xerostomia over time. Prevalence of xerostomia as reported in 14 studies represented by different colors, including both autologous and allogeneic HSCT recipients. The size of the circles refers to the quality of the reported outcome (the larger the circle, the lower the risk of bias). Proportion of allogeneic HSCT recipients per time period is listed below the graph. The gray dotted line represents the prevalence of xerostomia in the general population [56]

Prevalence of xerostomia over time after HSCT is shown in Fig. 2. Prevalence was based on questions using a yes/no format or ordinal questionnaires. The majority of the studies included both autologous and allogeneic HSCT recipients. Notwithstanding the heterogeneity between the studies, a clear trend over time is visible. Shortly after treatment, the prevalence of xerostomia increases, affecting the majority of patients during hospitalization. The prevalence starts to decline again after discharge, reaching levels largely comparable to baseline 1–2 years post-HSCT. Two studies could not be included in this figure, because results were reported as “ > 1 year post-HSCT,” which overlaps two of the chosen time periods: reported prevalences were 47% [53] and 49% [37]. The prevalence of xerostomia in HSCT recipients was high compared to the mean prevalence in the general population, as derived from a meta-analysis, including results of 26 epidemiological studies of adult, mostly older, populations [56].

### Severity of xerostomia

**Fig. 3:**
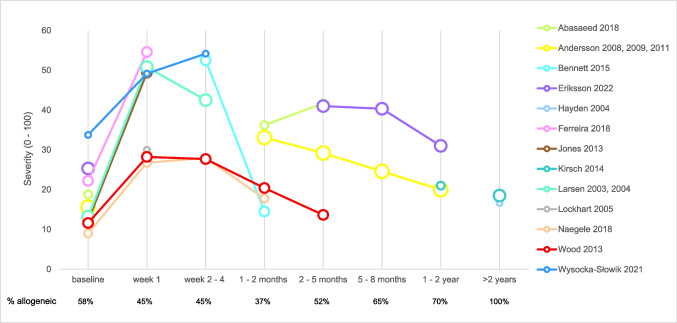
Severity of xerostomia over time. Rescaled severity of xerostomia (0–100) as reported in 13 studies represented by different colors, including both autologous and allogeneic HSCT recipients. The size of the circles refers to the quality of the reported outcome (the larger the circle, the lower the risk of bias). Proportion of allogeneic HSCT recipients per time period is listed below the graph

The severity of xerostomia over time after HSCT is shown in Fig. 3. The scores were derived from different questionnaires and recalculated to a scale from 0 to 100. The phrasing of the questions and the response options can be found in Table S5a.

Complaints increased shortly after the conditioning regimen: the increase in rescaled severity of xerostomia in the first week post-HSCT was on average 28 points (95% CI: 20–37), based on outcomes with a moderate risk of bias as reported in five studies (Figure S1a). Two to 5 months post-HSCT, severity of xerostomia was still raised with 13 points compared to baseline (95% CI: 7–18) based on outcomes with a moderate to low risk of bias as reported in four studies (Figure S1b). One to 2 years post-HSCT, the severity of xerostomia decreased further, reaching a level close to baseline (MD: 6, 95%CI: 2–10, moderate to low risk of bias, two studies, Figure S1c). No evidence was available to support a further decrease on the very long term.

### Distress caused by xerostomia

**Fig. 4:**
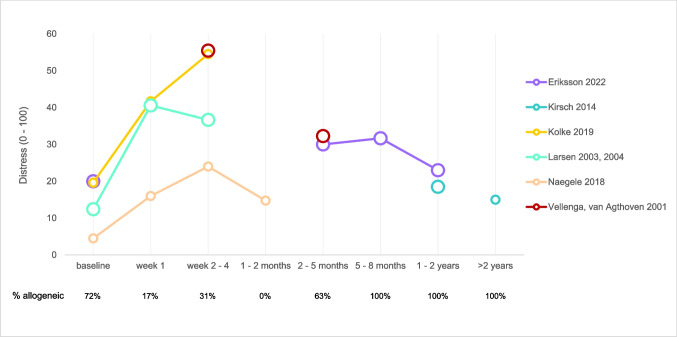
Distress caused by xerostomia over time. Rescaled distress (0–100) caused by xerostomia as reported in 6 studies represented by different colors, including both autologous and allogeneic HSCT recipients. The size of the circles refers to the quality of the reported outcome (the larger the circle, the lower the risk of bias). Proportion of allogeneic HSCT recipients per time period is listed below the graph

Only seven studies reported distress caused by xerostomia after HSCT. Results are shown in Fig. 4. The scores were derived from different questionnaires and recalculated to a scale from 0 to 100. The phrasing of the questions and the response options can be found in Table S5b. The distress caused by xerostomia increased shortly after treatment and decreases in the long term. Results of Warchala et al. (2019) [52] could not be included in this figure because it was unclear if discharge took place within 2–4 weeks post-HSCT [52]: the rescaled mean distress score was 23 at discharge.

### Risk indicators for the development of xerostomia

Risk indicators were defined to determine the influence of type of transplantation and conditioning regimen on the development of xerostomia. Severity data of autologous recipients, allogeneic recipients receiving MAC, and those receiving RIC could be reported separately. Such a distinction could not be made for prevalence and distress data. Limited data on the other risk indicators, type of conditioning regimen and development of cGvHD, was described. Even though risk indicators are discussed separately below, these factors are not independent.

### Type of HSCT and intensity of the conditioning regimen

**Fig. 5:**
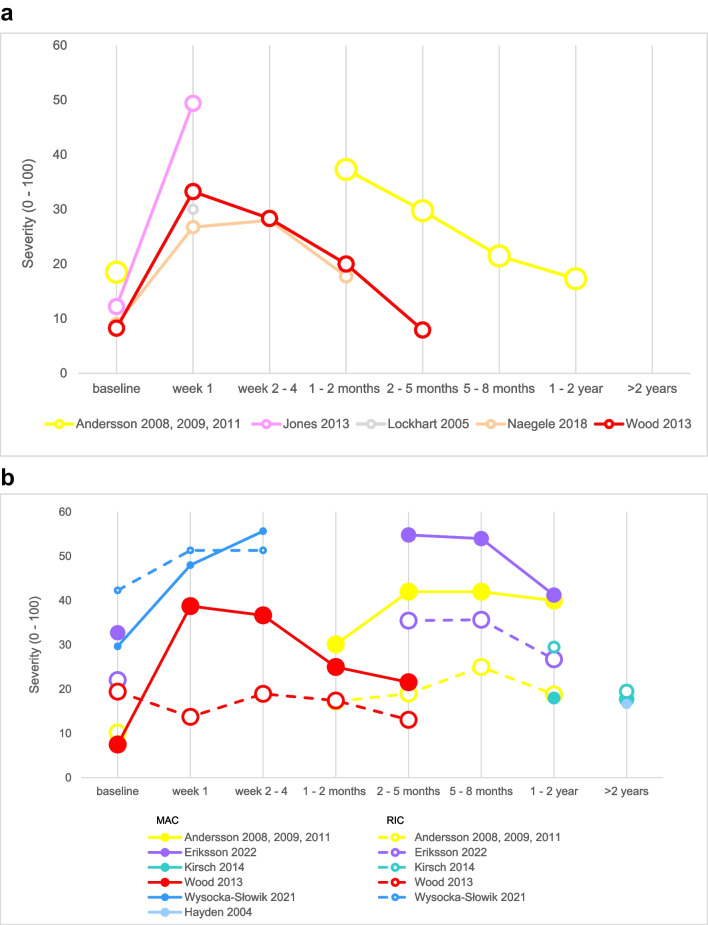
**a** Severity of xerostomia in autologous HSCT recipients. Rescaled severity of xerostomia (0–100) in autologous HSCT recipients as reported in 5 studies, represented by different colors. The size of the circles refers to the quality of the reported outcome (the larger the circle, the lower the risk of bias). **b** Severity of xerostomia in allogeneic HSCT recipients. Rescaled severity of xerostomia (0–100) in allogeneic HSCT recipients as reported in 6 studies, represented by different colors. Patients receiving a myeloablative conditioning regimen (MAC) are represented by solid lines and circles; patients receiving a reduced intensity conditioning regimen (RIC) are represented by dashed lines and empty circles. The size of the circles refers to the quality of the underlying subgroup (the larger the circle, the lower the risk of bias)

Three studies reported results for autologous and allogeneic recipients separately [23, 53, 54], and two studies divided allogeneic MAC and allogeneic RIC recipients [5, 55]. This distinction could be made for two other studies because individual patient data was provided [39, 44]. The severity of xerostomia in autologous HSCT recipients is shown in Fig. 5a and in allogeneic recipients in Fig. 5b.

In autologous HSCT recipients, the severity of xerostomia increased shortly after treatment; the decline started after discharge and resulted 1 year post-HSCT in levels similar to baseline. The severity of xerostomia in autologous recipients and allogeneic MAC recipients was comparable during the first week [54] and the first month post-HSCT [23, 54]. One year post-HSCT, allogeneic MAC recipients perceived more severe xerostomia than autologous HSCT recipients [23]. Furthermore, the prevalence of xerostomia > 1 year post-HSCT was 54% in allogeneic MAC recipients, while it was 39% in autologous recipients [53]. Following RIC, only a limited or even no increase in xerostomia was shown in the post-discharge period.

Looking at the allogeneic subgroups, it is shown that the heterogeneity between the studies at baseline was substantial. All meta-analysis performed to compare MAC and RIC included three studies reporting outcomes with a moderate risk of bias. There was no difference in the severity scores at baseline, comparing patients planned to receive MAC or RIC (MD: 0, 95% CI: − 13–12, Figure S2a). If the two groups were compared 2–5 months post-HSCT, MAC caused more severe xerostomia than RIC: the severity in MAC recipients was 18 points higher (95% CI: 9–27) compared to RIC recipients (Figure S2b). One to 2 years post-HSCT, the difference was not significant anymore (MD: 9, 95%CI: − 8–26, Figure S2c).

### Development of cGvHD

In cross-sectional studies with a long-term follow-up (> 1 year), the incidence of cGvHD in allogeneic HSCT recipients was 45% [37, 44], 48% [41], 54% [38], 59% [19], and 72% [6]. In longitudinal studies, the incidence of cGvHD was 39% [5] in the first year after treatment, and 27% 1 year post-HSCT [39]. None of the included studies determined the relation between cGvHD and xerostomia. Based on the rescaled individual patient data from Kirsch et al. (2014), patients with cGvHD had a higher prevalence of xerostomia (47%, mean severity: 25) in comparison to those without cGvHD (31%, mean severity: 14) [44].

### Type of conditioning regimen: chemotherapy with or without TBI

Looking at patients included in the subgroups (Fig. 5a, b), 1% of the autologous, 25% of the allogeneic RIC recipients, and 56% of the allogeneic MAC recipients received TBI (proportion of TBI recipients was unclear in one study [55]). Iestra et al. (2002) reported that TBI recipients experienced median 4 (range: 0–4) episodes of xerostomia in the first year post-HSCT, while patients treated without TBI experienced median 0 (range: 0–2) episodes [42]. Individual patient data from Eriksson et al. (2022) showed that TBI recipients perceived non-significantly more xerostomia during the first year after treatment, in comparison to those that did not receive TBI [39]. From Kirsch et al. (2014) individual patient data, it could be calculated that the prevalence of xerostomia between patients that received TBI (38%, mean severity 19) and those that did not receive TBI (37%, mean severity 17) did not differ > 1 year post-HSCT [44].

## Discussion

From this systematic review, it becomes clear that the prevalence of xerostomia in HSCT recipients is high in comparison to the general population [56]. This difference already exists at baseline, increases after HSCT, and remains after recovery in the long term. Based on reported outcomes with a moderate risk of bias, the severity of xerostomia increases shortly after HSCT with 28 points on a 0–100 scale (95% CI: 20–37) and declines after discharge from the hospital. We conclude that the severity of xerostomia is still raised 2–5 months post-HSCT and reaches scores largely comparable to baseline levels after 1 year. Distress caused by xerostomia follows the same trend over time.

The development of xerostomia is related to the decline in salivary flow rate after HSCT [20]. Comparing xerostomia with reduction in salivary flow rate, a comparable trend over time is seen. Stimulated flow rate decreases shortly after HSCT [24, 40], is still lowered 6 months post-HSCT [57, 58], and returns to baseline levels after 1 year [57, 58]. The reduction in salivary flow rate is predominately attributed to the conditioning regimen toxicity and the high number of medications that the patients use preceding and following the transplantation [9].

The reported prevalence of xerostomia is much higher than the prevalence of hyposalivation [9, 12, 19]; thus, xerostomia could only partially be explained by a reduction in flow rate. Change in composition [20] and viscosity [59] of saliva, or neuropathic mechanisms [18], might be related to xerostomia as well. Furthermore, we hypothesize that xerostomia might be associated with changes in mucosa due to mucositis or cGvHD. Another risk indicator for the development of xerostomia might be the psychological distress that the patients experience in relation to the disease and the treatment [26, 60].

The intensity of the conditioning regimen is the most objectifiable risk indicator for the development of xerostomia. Severity of xerostomia shows a marked increase in the two subgroups receiving a high intensity conditioning regimen, but not in the subgroup receiving RIC. Higher intensity conditioning is also related to a higher incidence of oral mucositis [61], a higher patient-reported symptom severity [62], and a tendency towards increasing prevalence of hyposalivation [57]. We conclude with a moderate risk of bias that severity of xerostomia is higher in allogeneic MAC recipients compared to RIC recipients 2–5 months post-HSCT (rescaled MD: 18; 95% CI: 9–27). This difference did not exist at baseline, and is not significant anymore after 1–2 years.

After conducting this systematic review, it remains unclear whether TBI has an additional effect on the development of xerostomia compared to chemotherapy alone. Even though patients who received TBI as part of the conditioning regimen had a higher risk of mouth dryness [63], other publications found no [8] or no significant additional effect of TBI [2]. The dose of TBI administered as part of the conditioning regimen might not reach the threshold above which salivary gland function will be diminished [64].

In the autologous subgroup, the severity of xerostomia starts to decline after discharge, reaching baseline levels after 3 or 12 months. The peak in complaints in allogeneic HSCT recipients seems to be delayed or prolonged, and baseline levels might not be reached after 1 year. The relatively high post-discharge severity of xerostomia in allogeneic HSCT recipients might be related to the development of cGvHD. cGvHD is associated with histopathological changes in salivary glands, a reduction in flow rate, and a change in the composition of saliva [65]. None of the included studies determined the relation between cGvHD and xerostomia, but a higher prevalence of xerostomia could be calculated in patients that developed cGvHD in comparison to those without cGvHD [44]. This finding is in agreement with the results of several studies that did not meet the inclusion criteria of this review [28, 66–68]. Even though a higher prevalence of xerostomia is reported > 1 year post-treatment in allogeneic compared to autologous HSCT recipients [53], older studies could not confirm this difference in the long term [69, 70].

The decrease in mouth dryness over time may be affected by loss to follow-up of the patients with the most complaints. Loss to follow-up in the populations is extensive and unavoidable, given that the mortality rate in the first year after treatment is 10% [23], 24% [39], and 25% [42]. Relapse (23% [39] and 32% [23] during the first year post-HSCT), worsening of physical condition, and psychological distress are other reasons for loss to follow-up. The prevalence of xerostomia is lower in study completers in comparison to those that will be lost to follow up in the first year after HSCT [42]. This difference was clear at any measurement timepoint.

The results of the current review should be interpreted with caution, because the variation between the studies is extensive, and HSCT recipients comprise heterogenous populations. The included studies are conducted in different geographic regions and included adult populations of different ages, which might have influenced perceived mouth dryness [56]. Furthermore, several questionnaires using different phrasing, different response options, and different time frames will have provided heterogeneity in the results. For example, studies using a 5- [49, 54] or 10-point scale [43] tend to report lower mean xerostomia scores at baseline than those using a 4-point scale [23, 34, 39, 40, 47, 55]. Besides, chosen thresholds to calculate the prevalence of xerostomia influence the results: Jones et al. [43] reported the prevalence of moderate/severe xerostomia, explaining the low prevalence in this study. Because of the large heterogeneity between the studies, conclusions about the development of xerostomia over time are only based on within study differences.

Despite the relatively strict inclusion criteria used in the current review, the number of included studies is high in comparison to the previous systematic review conducted in 2010 [1]. This high number of included studies can be attributed to the extensive and detailed search strategy. Since xerostomia is reported in most papers as secondary outcome, or as part of a questionnaire assessing symptoms or quality of life, the terms “xerostomia” or “mouth dryness” were not reported in titles and rarely in abstracts. None of the included studies used a questionnaire specifically developed to measure the severity of xerostomia [71]. A disadvantage of this approach is that the outcome xerostomia itself is not validated, although it is frequently extracted from validated questionnaires. Even though mean severity or distress scores are not the optimal way of reporting xerostomia, since the underlying data will not be normally distributed, we judged that mean scores are the best outcome to visualize changes over time.

Risk of bias was assessed with the quality grading strategy published by Brennen et al. in 2010 [32], aiming to rate the risk of bias of oral complications from cancer therapies. This strategy was chosen because it matched our research question very well. Another advantage of this approach is that the risk of bias of the xerostomia outcomes was rated instead of the overall study quality, which is more appropriate because the majority of the studies was not designed to report specifically on oral complications. Use of questionnaires that were not validated to measure xerostomia and inclusion of relatively small single-center populations resulted in reduced quality scores for the xerostomia outcome (median 4, range 1–7). The meta-analysis, conducted to support conclusions drawn from the graphs, includes only studies that reported xerostomia with a moderate to low risk of bias to increase their robustness [72].

Patient-reported subjective mouth dryness or xerostomia is a serious complaint after HSCT affecting the majority of the patients. Patient-reported outcome measures are associated with quality of life measures [73] and reflect daily health status better than clinician reported outcomes [74]. Xerostomia is even rated as one of the most bothersome symptoms by patients [5, 6]. Complaints in autologous HSCT recipients seems to be transient in nature, while severity of xerostomia in allogeneic MAC recipients might remain elevated. The intensity of the conditioning plays a key role in the short-term development of xerostomia, while factors affecting the long-term recovery remain largely unknown. Further longitudinal studies are warranted, focusing on the effect of TBI, medications, and development of cGvHD as potential risk indicators for xerostomia.

## Supplementary information

Below is the link to the electronic supplementary material.

Supplementary file1 (DOCX 243 KB)

## References

[1] Jensen SB, Pedersen AM, Vissink A, Andersen E, Brown CG, Davies AN, Dutilh J, Fulton JS, Jankovic L, Lopes NN, Mello AL, Muniz LV, Murdoch-Kinch CA, Nair RG, Napeñas JJ, Nogueira-Rodrigues A, Saunders D, Stirling B, von Bültzingslöwen I, Weikel DS, Elting LS, Spijkervet FK, Brennan MT (2010). A systematic review of salivary gland hypofunction and xerostomia induced by cancer therapies: prevalence, severity and impact on quality of life. Support Care Cancer.

[2] Brand HS, Bots CP, Raber-Durlacher JE (2009). Xerostomia and chronic oral complications among patients treated with haematopoietic stem cell transplantation. Br Dent J.

[3] Tanasiewicz M, Hildebrandt T, Obersztyn I (2016). Xerostomia of various etiologies: a review of the literature. Adv Clin Exp Med.

[4] Sreebny LM, Valdini A (1988). Xerostomia. 1. Relationship to other oral symptoms and salivary-gland hypofunction. Oral Surg Oral Med O.

[5] Andersson I, Ahlberg K, Stockelberg D, Brune M, Persson LO (2009). Health-related quality of life in patients undergoing allogeneic stem cell transplantation after reduced intensity conditioning versus myeloablative conditioning. Cancer Nurs.

[6] Edman L, Larsen J, Hägglund H, Gardulf A (2001). Health-related quality of life, symptom distress and sense of coherence in adult survivors of allogeneic stem-cell transplantation. Eur J Cancer Care (Engl).

[7] Bos-den Braber J, Potting CM, Bronkhorst EM, Huysmans MC, Blijlevens NM (2015). Oral complaints and dental care of haematopoietic stem cell transplant patients: a qualitative survey of patients and their dentists. Support Care Cancer.

[8] Hull KM, Kerridge I, Schifter M (2012). Long-term oral complications of allogeneic haematopoietic SCT. Bone Marrow Transplant.

[9] Boer CC, Correa ME, Miranda EC, de Souza CA (2010). Taste disorders and oral evaluation in patients undergoing allogeneic hematopoietic SCT. Bone Marrow Transplant.

[10] Thomson WM, Chalmers JM, Spencer AJ, Williams SM (1999). The Xerostomia Inventory: a multi-item approach to measuring dry mouth. Community Dent Health.

[11] Hjermstad MJ, Bergenmar M, Bjordal K, Fisher SE, Hofmeister D, Montel S, Nicolatou-Galitis O, Pinto M, Raber-Durlacher J, Singer S, Tomaszewska IM, Tomaszewski KA, Verdonck-de Leeuw I, Yarom N, Winstanley JB, Herlofson BB, Group EQ (2016). International field testing of the psychometric properties of an EORTC quality of life module for oral health: the EORTC QLQ-OH15. Support Care Cancer.

[12] Daikeler T, Mauramo M, Rovó A, Stern M, Halter J, Buser A, Tyndall A, Häusermann P, Gratwohl A, Tichelli A, Brennan MT, Waltimo T (2013). Sicca symptoms and their impact on quality of life among very long-term survivors after hematopoietic SCT. Bone Marrow Transplant.

[13] Bassim CW, Fassil H, Mays JW, Edwards D, Baird K, Steinberg SM, Cowen EW, Naik H, Datiles M, Stratton P, Gress RE, Pavletic SZ (2015). Oral disease profiles in chronic graft versus host disease. J Dent Res.

[14] Fox PC, Busch KA, Baum BJ (1987). Subjective reports of xerostomia and objective measures of salivary gland performance. J Am Dent Assoc.

[15] Wolff M, Kleinberg I (1998). Oral mucosal wetness in hypo- and normosalivators. Arch Oral Biol.

[16] Dawes C, Odlum O (2004). Salivary status in patients treated for head and neck cancer. J Can Dent Assoc.

[17] Pedersen AML, Sorensen CE, Proctor GB, Carpenter GH, Ekstrom J (2018). Salivary secretion in health and disease. J Oral Rehabil.

[18] da Travassos Rosa Moreira Bastos R, Mecenas P, Normando D (2021). Effects of dietary consistency on the occlusal changes in nonhuman mammals: a systematic review. Arch Oral Biol.

[19] Arduino PG, Gambino A, Giaccone L, Suria M, Carbone M, Carrozzo M, Broccoletti R, Conrotto D (2022). Oral health status after hematopoietic stem cell transplantations: outcomes from an adult Italian population. Spec Care Dentist.

[20] van Leeuwen SJM, Potting CMJ, Huysmans M, Blijlevens NMA (2019). Salivary changes before and after hematopoietic stem cell transplantation: a systematic review. Biol Blood Marrow Transplant.

[21] Buchali A, Feyer P, Groll J, Massenkeil G, Arnold R, Budach V (2000). Immediate toxicity during fractionated total body irradiation as conditioning for bone marrow transplantation. Radiother Oncol.

[22] Chaillet MP, Cosset JM, Socie G, Pico JL, Grimaud E, Dubray B, Alapetite C, Girinsky T (1993). Prospective study of the clinical symptoms of therapeutic whole body irradiation. Health Phys.

[23] Andersson I, Ahlberg K, Stockelberg D, Persson LO (2011). Patients’ perception of health-related quality of life during the first year after autologous and allogeneic stem cell transplantation. Eur J Cancer Care (Engl).

[24] Chaushu G, Itzkovitz-Chaushu S, Yefenof E, Slavin S, Or R, Garfunkel AA (1995). A longitudinal follow-up of salivary secretion in bone marrow transplant patients. Oral Surg Oral Med Oral Pathol Oral Radiol Endod.

[25] Wolff A, Joshi RK, Ekstrom J, Aframian D, Pedersen AM, Proctor G, Narayana N, Villa A, Sia YW, Aliko A, McGowan R, Kerr AR, Jensen SB, Vissink A, Dawes C (2017). A guide to medications inducing salivary gland dysfunction, xerostomia, and subjective sialorrhea: a systematic review sponsored by the world workshop on oral medicine VI. Drugs R D.

[26] Abel GA, Albelda R, Khera N, Hahn T, Coronado DYS, Odejide OO, Bona K, Tucker-Seeley R, Soiffer R (2016). Financial hardship and patient-reported outcomes after hematopoietic cell transplantation. Biol Blood Marrow Tr.

[27] Bulthuis MS, Jan Jager DH, Brand HS (2018). Relationship among perceived stress, xerostomia, and salivary flow rate in patients visiting a saliva clinic. Clin Oral Investig.

[28] Gomes AO, Torres SR, Maiolino A, Dos Santos CW, Silva Junior A, Correa ME, Moreira MC, Goncalves Lde S (2014). Early and late oral features of chronic graft-versus-host disease. Rev Bras Hematol Hemoter.

[29] Page MJ, McKenzie JE, Bossuyt PM, Boutron I, Hoffmann TC, Mulrow CD, Shamseer L, Tetzlaff JM, Akl EA, Brennan SE, Chou R, Glanville J, Grimshaw JM, Hrobjartsson A, Lalu MM, Li T, Loder EW, Mayo-Wilson E, McDonald S, McGuinness LA, Stewart LA, Thomas J, Tricco AC, Welch VA, Whiting P, Moher D (2021). The PRISMA 2020 statement: an updated guideline for reporting systematic reviews. BMJ.

[30] Cox JD, Stetz J, Pajak TF (1995). Toxicity criteria of the Radiation Therapy Oncology Group (RTOG) and the European Organization for Research and Treatment of Cancer (EORTC). Int J Radiat Oncol Biol Phys.

[31] Cancer Therapy Evaluation Program, Common Terminology Criteria for Adverse Events, Version 5.0. (2017). http://ctep.cancer.gov

[32] Brennan MT, Elting LS, Spijkervet FKL (2010). Systematic reviews of oral complications from cancer therapies, Oral Care Study Group, MASCC/ISOO: methodology and quality of the literature. Support Care Cancer.

[33] Higgins JPT, Thomas J, Chandler J, Cumpston M, Li T, Page MJ, Welch VA (eds)(2022) Cochrane handbook for systematic reviews of interventions version 6.3 (updated February 2022). Cochrane. Available from https://www.training.cochrane.org/handbook 10.1002/14651858.ED000142 PMC10284251 31643080

[34] Abasaeed R, Coldwell SE, Lloid ME, Soliman SH, Macris PC, Schubert MM (2018). Chemosensory changes and quality of life in patients undergoing hematopoietic stem cell transplantation. Support Care Cancer.

[35] Andersson I, Hjermstad M, Stockelberg D, Persson LO (2008). Health related quality of life in stem cell transplantation: clinical and psychometric validation of the questionnaire module, High Dose Chemotherapy (HDC-19). Acta Oncol.

[36] Bennett AV, Reeve BB, Basch EM, Mitchell SA, Meeneghan M, Battaglini CL, Smith-Ryan AE, Phillips B, Shea TC, Wood WA (2016). Evaluation of pedometry as a patient-centered outcome in patients undergoing hematopoietic cell transplant (HCT): a comparison of pedometry and patient reports of symptoms, health, and quality of life. Qual Life Res.

[37] Bergkvist K, Winterling J, Johansson E, Johansson UB, Svahn BM, Remberger M, Mattsson J, Larsen J (2015). General health, symptom occurrence, and self-efficacy in adult survivors after allogeneic hematopoietic stem cell transplantation: a cross-sectional comparison between hospital care and home care. Support Care Cancer.

[38] Cheon J, Lee YJ, Jo JC, Kweon K, Koh S, Min YJ, Park SH, Lee SH, Kim HJ, Choi Y (2021). Late complications and quality of life assessment for survivors receiving allogeneic hematopoietic stem cell transplantation. Support Care Cancer.

[39] Eriksson LV, Holmberg K, Lundh Hagelin C, Wengström Y, Bergkvist K, Winterling J (2022). Symptom burden and recovery in the first year after allogeneic hematopoietic stem cell transplantation. Cancer Nurs.

[40] Ferreira MH, Mello Bezinelli L, de Paula EF, Lopes RM, Pereira AZ, Hamerschlack N, Corrêa L (2020). Association of oral toxicity and taste changes during hematopoietic stem cell transplantation: a preliminary study. Support Care Cancer.

[41] Hayden PJ, Keogh F, Ni Conghaile M, Carroll M, Crowley M, Fitzsimon N, Gardiner N, Vandenberghe E, O’Riordan J, McCann SR (2004). A single-centre assessment of long-term quality-of-life status after sibling allogeneic stem cell transplantation for chronic myeloid leukaemia in first chronic phase. Bone Marrow Transplant.

[42] Iestra JA, Fibbe WE, Zwinderman AH, van Staveren WA, Kromhout D (2002). Body weight recovery, eating difficulties and compliance with dietary advice in the first year after stem cell transplantation: a prospective study. Bone Marrow Transplant.

[43] Jones D, Vichaya EG, Wang XS, Williams LA, Shah ND, Thomas SK, Johnson VE, Champlin RE, Cleeland CS, Mendoza TR (2013). Validation of the M. D. Anderson Symptom Inventory multiple myeloma module. J Hematol Oncol.

[44] Kirsch M (2014) Patient reported outcomes in view of symptom experience of late effects and self-management of adult long-term survivors after allogeneic haematopoietic stem cell transplantation - a mixed methods study. Published doctoral dissertation, University of Basel, Basel

[45] Kolke S, Kuhlenschmidt M, Bauer E, Anthony MK, Gittleman H, Caimi PF, Mazanec SR (2019). Factors influencing patients’ intention to perform physical activity during hematopoietic cell transplantation. Oncol Nurs Forum.

[46] Larsen J, Nordström G, Björkstrand B, Ljungman P, Gardulf A (2003). Symptom distress, functional status and health-related quality of life before high-dose chemotherapy with stem-cell transplantation. Eur J Cancer Care (Engl).

[47] Larsen J, Nordström G, Ljungman P, Gardulf A (2004). Symptom occurrence, symptom intensity, and symptom distress in patients undergoing high-dose chemotherapy with stem-cell transplantation. Cancer Nurs.

[48] Lockhart PB, Brennan MT, Kent ML, Packman CH, Norton HJ, Fox PC, Frenette G (2005). Randomized controlled trial of pilocarpine hydrochloride for the moderation of oral mucositis during autologous blood stem cell transplantation. Bone Marrow Transplant.

[49] Naegele M, Kirsch M, Ihorst G, Fierz K, Engelhardt M, De Geest S (2018). Symptom experience of multiple myeloma (syMMex) patients treated with autologous stem cell transplantation following high-dose melphalan: a descriptive longitudinal study. Support Care Cancer.

[50] Vellenga E, van Agthoven M, Croockewit AJ, Verdonck LF, Wijermans PJ, van Oers MH, Volkers CP, van Imhoff GW, Kingma T, Uyl-de Groot CA (2001). Autologous peripheral blood stem cell transplantation in patients with relapsed lymphoma results in accelerated haematopoietic reconstitution, improved quality of life and cost reduction compared with bone marrow transplantation: the Hovon 22 study. Br J Haematol.

[51] van Agthoven M, Vellenga E, Fibbe WE, Kingma T, Uyl-de Groot CA (2001). Cost analysis and quality of life assessment comparing patients undergoing autologous peripheral blood stem cell transplantation or autologous bone marrow transplantation for refractory or relapsed non-Hodgkin’s lymphoma or Hodgkin’s disease. a prospective randomised trial. Eur J Cancer.

[52] Warchala A, Krupka-Matuszczyk I, Krysta K (2019). Anxiety and depression in patients with acute leukaemia treated with hematopoietic stem cell transplantation. Psychiatr Danub.

[53] Watson M, Buck G, Wheatley K, Homewood JR, Goldstone AH, Rees JK, Burnett AK (2004). Adverse impact of bone marrow transplantation on quality of life in acute myeloid leukaemia patients; analysis of the UK Medical Research Council AML 10 Trial. Eur J Cancer.

[54] Wood WA, Deal AM, Abernethy A, Basch E, Battaglini C, Kim YH, Whitley J, Shatten C, Serody J, Shea T, Reeve BB (2013). Feasibility of frequent patient-reported outcome surveillance in patients undergoing hematopoietic cell transplantation. Biol Blood Marrow Transplant.

[55] Wysocka-Slowik A, Gil L, Slebioda Z, Dorocka-Bobkowska B (2021). Oral complaints in patients with acute myeloid leukemia treated with allogeneic hematopoietic stem cell transplantation. Medicina Oral Patologia Oral y Cirugia Bucal.

[56] Agostini BA, Cericato GO, Silveira ERD, Nascimento GG, Costa FDS, Thomson WM, Demarco FF (2018). How common is dry mouth? Systematic review and meta-regression analysis of prevalence estimates. Braz Dent J.

[57] Laaksonen M, Ramseier AM, Rovó A, Jensen SB, Raber-Durlacher JE, Zitzmann NU, Waltimo T (2011). Longitudinal assessment of hematopoietic stem cell transplantation and hyposalivation. J Dent Res.

[58] Uutela P, Passweg J, Halter J, Gerull S, Weiger R, Mauramo E, Waltimo T, Mauramo M (2019). Common oral diseases, hyposalivation and survival post-HSCT, a longitudinal study. Eur J Haematol.

[59] Kolbinson DA, Schubert MM, Flournoy N, Truelove EL (1988). Early oral changes following bone marrow transplantation. Oral Surg Oral Med Oral Pathol.

[60] Kuba K, Esser P, Scherwath A, Schirmer L, Schulz-Kindermann F, Dinkel A, Balck F, Koch U, Kroger N, Gotze H, Mehnert A (2017). Cancer-and-treatment-specific distress and its impact on posttraumatic stress in patients undergoing allogeneic hematopoietic stem cell transplantation (HSCT). Psychooncology.

[61] Ringden O, Erkers T, Aschan J, Garming-Legert K, Le Blanc K, Hagglund H, Omazic B, Svenberg P, Dahllof G, Mattsson J, Ljungman P, Remberger M (2013). A prospective randomized toxicity study to compare reduced-intensity and myeloablative conditioning in patients with myeloid leukaemia undergoing allogeneic haematopoietic stem cell transplantation. J Intern Med.

[62] Cohen MZ, Rozmus CL, Mendoza TR, Padhye NS, Neumann J, Gning I, Aleman A, Giralt S, Cleeland CS (2012). Symptoms and quality of life in diverse patients undergoing hematopoietic stem cell transplantation. J Pain Symptom Manage.

[63] Majhail NS, Ness KK, Burns LJ, Sun CL, Carter A, Francisco L, Forman SJ, Bhatia S, Baker KS (2007). Late effects in survivors of Hodgkin and non-Hodgkin lymphoma treated with autologous hematopoietic cell transplantation: a report from the bone marrow transplant survivor study. Biol Blood Marrow Transplant.

[64] Mercadante V, Jensen SB, Smith DK, Bohlke K, Bauman J, Brennan MT, Coppes RP, Jessen N, Malhotra NK, Murphy B, Rosenthal DI, Vissink A, Wu J, Saunders DP, Peterson DE (2021). Salivary gland hypofunction and/or xerostomia induced by nonsurgical cancer therapies: ISOO/MASCC/ASCO Guideline. J Clin Oncol.

[65] Nagler RM, Nagler A (2004). Salivary gland involvement in graft-versus-host disease: the underlying mechanism and implicated treatment. Isr Med Assoc J.

[66] Schubert MM, Sullivan KM, Morton TH, Izutsu KT, Peterson DE, Flournoy N, Truelove EL, Sale GE, Buckner CD, Storb R (1984). Oral manifestations of chronic graft-v-host disease. Arch Intern Med.

[67] Nagler R, Marmary Y, Krausz Y, Chisin R, Markitziu A, Nagler A (1996). Major salivary gland dysfunction in human acute and chronic graft-versus-host disease (GVHD). Bone Marrow Transplant.

[68] Lenssen P, Sherry ME, Cheney CL, Nims JW, Sullivan KM, Stern JM, Moe G, Aker SN (1990). Prevalence of nutrition-related problems among long-term survivors of allogeneic marrow transplantation. J Am Diet Assoc.

[69] Hjermstad M, Holte H, Evensen S, Fayers P, Kaasa S (1999). Do patients who are treated with stem cell transplantation have a health-related quality of life comparable to the general population after 1 year?. Bone Marrow Transplant.

[70] Zittoun R, Suciu S, Watson M, Solbu G, Muus P, Mandelli F, Stryckmans P, Peetermans M, Thaler J, Resegotti L, Dardenne M, Willemze R (1997). Quality of life in patients with acute myelogenous leukemia in prolonged first complete remission after bone marrow transplantation (allogeneic or autologous) or chemotherapy: a cross-sectional study of the EORTC-GIMEMA AML 8A trial. Bone Marrow Transplant.

[71] Assas M, Wiriyakijja P, Fedele S, Porter S, Ni Riordain R (2021). Evaluating the measurement properties of patient-reported outcome measures in radiotherapy-induced xerostomia. Oral Dis.

[72] Carroll C, Booth A, Lloyd-Jones M (2012). Should we exclude inadequately reported studies from qualitative systematic reviews? An evaluation of sensitivity analyses in two case study reviews. Qual Health Res.

[73] Pidala J, Kurland BF, Chai X, Vogelsang G, Weisdorf DJ, Pavletic S, Cutler C, Majhail N, Lee SJ (2011). Sensitivity of changes in chronic graft-versus-host disease activity to changes in patient-reported quality of life: results from the Chronic Graft-versus-Host Disease Consortium. Haematologica.

[74] Basch E, Jia X, Heller G, Barz A, Sit L, Fruscione M, Appawu M, Iasonos A, Atkinson T, Goldfarb S, Culkin A, Kris MG, Schrag D (2009). Adverse symptom event reporting by patients vs clinicians: relationships with clinical outcomes. J Natl Cancer Inst.

