# Supplementary material for: The effect of hematopoietic stem cell transplantation on patient-reported subjective oral dryness: a systematic review focusing on prevalence, severity and distress
Source: Support Care Cancer. 2023 Jul 8;31(8):449. doi: 10.1007/s00520-023-07921-1 (PMC10329604; doi:10.1007/s00520-023-07921-1)
Supplement: Supplementary file 1 — Supplementary file1 (DOCX 243 KB) [file 520_2023_7921_MOESM1_ESM.docx]

**Table S1a, Cochrane Central Register of Controlled Trials (CENTRAL) search strategy**

| #1 | MeSH descriptor: [Bone Marrow Transplantation] explode all trees |
| --- | --- |
| #2 | (((bone marrow or cord blood or cord stem or marrow stem or placental blood or peripheral blood) and (graft* OR transplant*)) or BMT or (stem cell and transplant*) or SCT or HSCT):ti,ab,kw |
| #3 | (Patient Reported Outcome* or PROM):ti,ab,kw |
| #4 | MeSH descriptor: [Xerostomia] explode all trees |
| #5 | (xerostomia or ((dry*or sicca) and (oral or mouth)) or (saliva* and (flow or secret*)) or hyposalivation):ti,ab,kw |
| #6 | MeSH descriptor: [Taste] this term only |
| #7 | MeSH descriptor: [Mouth] explode all trees |
| #8 | MeSH descriptor: [Oral Manifestations] explode all trees |
| #9 | MeSH descriptor: [Saliva] explode all trees |
| #10 | (Oral or taste or tastes or mouth or mouths or saliva* or dentition or questionnaire):ti,ab,kw |
| #11 | MeSH descriptor: [Surveys and Questionnaires] explode all trees |
| #12 | MeSH descriptor: [Quality of Life] explode all trees |
| #13 | (Quality of life or HRQOL or QoL):ti,ab,kw |
| #14 | (MSAS or SFID or QLQ or RSCL or PedsQl or “medical late effects” or “Physical Well Being” or oral complaint* or "oral adverse effects" or “oral side effects” or “oral side effect” or "oral toxicity" or “oral discomfort” or “Symptom scale” or “Symptom Checklist” or "symptom assessment"):ti,ab,kw |
| #15 | #1 or #2 |
| #16 | #6 or #7 or #8 or #9 or #10 or #11 |
| #17 | #12 OR #13 |
| #18 | #16 and #17 |
| #19 | #3 or #4 or #5 |
| #20 | #18 or #19 or #14 |
| #21 | #15 and #20 |

**Table S1b, MEDLINE (Pubmed) search strategy**

| 1: "bone marrow transplantation"[Mesh] OR (("bone marrow"[Tiab] OR cord blood[tiab] OR cord stem[tiab] OR marrow stem[tiab] OR placental blood[tiab] OR peripheral blood[tiab]) AND (graft*[Tiab] OR transplant*[Tiab])) OR BMT[Tiab] OR “stem cell transplantation”[Mesh] OR (“stem cell”[Tiab] AND transplant*[Tiab]) OR SCT[Tiab] OR HSCT[Tiab] |
| --- |
| 2: "Patient Reported Outcome Measures "[Mesh:noexp] OR PROM[tiab] OR patient reported outcome*[tiab] |
| 3: xerostomia[Mesh:noexp] OR xerostomia[Tiab] OR (dry*[Tiab] OR sicca[Tiab]) AND (oral[Tiab] OR mouth[tiab]) |
| 4: saliva*[Tiab] AND (flow[Tiab] or secret*Tiab])) OR hyposalivation[Tiab] |
| 5: "Taste"[Mesh] OR "Mouth"[Mesh] OR "Oral Manifestations"[Mesh] OR "Saliva"[Mesh] OR Oral[tiab] OR Taste[tiab] OR Tastes[tiab] OR Mouth[tiab] OR Mouths[tiab] OR Saliva*[tiab] OR Dentition[tiab] |
| 6: "Dental Health Surveys"[Mesh:noexp] OR "Patient Health Questionnaire"[Mesh] OR "Self Report"[Mesh] OR questionnaire[Tiab] OR “Symptom scale”[Tiab] |
| 7:“Quality of life”[Mesh] OR Quality of life[Tiab] OR HRQOL[Tiab] OR QoL[tiab] |
| 8: (5 OR 6) AND 7 |
| 9: MSAS[Tiab] OR SFID[Tiab] OR QLQ[Tiab] OR RSCL[Tiab] OR PedsQl[Tiab] |
| 10: “medical late effects”[Tiab] OR “Physical Well Being”[Tiab] OR oral complaint*[tiab] OR "oral adverse effects"[Tiab] OR “oral side effects”[tiab] OR “oral side effect”[tiab] OR "oral toxicity"[Tiab] OR “oral discomfort”[Tiab] OR “Symptom scale”[Tiab] OR “Symptom Checklist”[Tiab] OR "symptom assessment"[Tiab] |
| 11: 2 OR 3 OR 4 OR 7 OR 8 OR 9 OR 10 |
| 12: 1 AND 11 |

**Table S1c, EMBASE (Ovid) search strategy**

| (exp bone marrow transplantation/ OR ((bone marrow.ti,ab,kw. OR cord blood.ti,ab,kw. OR cord stem.ti,ab,kw. OR marrow stem.ti,ab,kw. OR placental blood.ti,ab,kw. OR peripheral blood.ti,ab,kw.) AND (graft*.ti,ab,kw. OR transplant*.ti,ab,kw.)) OR BMT.ti,ab,kw. OR stem cell transplantation/ or exp allogeneic stem cell transplantation/ or exp autologous stem cell transplantation/ or cord blood stem cell transplantation/ or exp hematopoietic stem cell transplantation/ or nonmyeloablative stem cell transplantation/ or exp peripheral blood stem cell transplantation/ OR (stem cell.ti,ab,kw. AND transplant*.ti,ab,kw.) OR SCT.ti,ab,kw. OR HSCT.ti,ab,kw.) |
| --- |
| AND (xerostomia/ OR xerostomia.ti,ab,kw. OR ((dry*.ti,ab,kw. OR sicca.ti,ab,kw.) AND (oral.ti,ab,kw. OR mouth.ti,ab,kw.)) OR (saliv*.ti,ab,kw. AND (flow.ti,ab,kw. or secret*.ti,ab,kw.)) OR hyposalivation.ti,ab,kw. OR MSAS.ti,ab,kw. OR SFID.ti,ab,kw OR medical late effects.ti,ab,kw. OR Physical Well Being.ti,ab,kw. OR oral complaint.ti,ab,kw. OR oral complaints.ti,ab,kw. OR oral adverse effects.ti,ab,kw. OR oral side effects.ti,ab,kw. OR oral side effect.ti,ab,kw.) OR ((Taste/ OR taste discrimination/ OR exp Mouth/ OR mouth tissue/ OR mouth discomfort/ or mouth injury/ or mouth lesion/ OR Saliva/ OR exp salivary gland/ OR exp taste disorder/ OR Oral.ti,ab,kw. OR Taste.ti,ab,kw. OR Tastes.ti,ab,kw. OR Mouth.ti,ab,kw. OR Mouths.ti,ab,kw. OR Saliva*.ti,ab,kw. OR Dentition.ti,ab,kw. OR symptom scales.ti,ab,kw.) AND ("quality of life"/ OR Quality of life.ti,ab,kw. OR HRQOL.ti,ab,kw. OR QoL.ti,ab,kw.)) |

**Table S2, Quality grading strategy for patient-reported mouth dryness, adapted from Brennan et al. (2010)**

| **Quality measures** | **Quality points and definitions** | **Adaptations and additions** |
| --- | --- | --- |
| Representativeness | 2: Multi-institution, consecutive* patients representative of underlying population  1: Single institution, consecutive patients, representative of underlying population  0: Convenience sample | Multi-institution, convenience sample was also classified as 1  * the word ‘consecutive’ should be reported or consecutive inclusion is likely based on described inclusion- and exclusion criteria, and numbers of excluded patients |
| Ascertainment bias | 2: >4 assessments before/after HSCT  1: 2 – 4 assessments before/after HSCT  0: 1 assessment before/after HSCT | Intervals as suggested for patients receiving radiotherapy were used (instead of chemotherapy as suggested by Brennan et al. 2010) |
| Misclassification bias | 1: Prospective (patient or professional)  0: Retrospective (patient recall) | Prospective: time frame of ≤1 week used to recall symptoms  Retrospective: time frame of >1 week used to recall symptoms |
| Examiner bias | 1: Blinded  0: Unblinded | Blinded: questionnaire filled out by the patient or interview by blinded examiner  Unblinded: interview by unblinded examiner or not reported |
| Oral complication assessment validity | 2: Standard validated scale  1: Well-defined, study-specific scale  0: Not defined | Only studies using a questionnaire validated to measure xerostomia received 2 points |
| Estimate precision | Sample size sufficient to estimate a prevalence of 20% within  2: ±5%  1: ±10%  0: Greater than 10% | 2: n >246  1: n 61 – 246  0: n < 61 |

**Table S3, characteristics of excluded studies**

Reasons for exclusion are reported in bold. If applicable, details are added in italics.

| **Data of HSCT recipients not reported separately** |
| --- |
| - Frowen et al 2020 (1)  - Gerecitano et al. 2011 (2)  - Hinkelmann et al. 2021 (3)  - Oerlemans et al. 2014 (4)  - Vadhan-Raj et al. 2010 (5) |
| **Time range too broad/unclear** |
| - Bos-den Braber et al. 2015 (6) *8 – 31 months post-HSCT*  - Brand et al. 2009 (7) *5.5 ± 4.9 years post HSCT*  - Caliscan et al. 2022(8) *16 – 70 days post-HSCT**  - Fantozzi et al. 2020 (9)  - Gomes et al. 2014 (10) *48 – 4100 days post-HSCT*  - Hull et al. 2012 (11) *6 months-6 years post HSCT*  - Kirsch et al. 2015 (12)  - Meirelles & Diez-Garcia 2018 (13) *15 – 65 days post-HSCT*  - Oguz et al 2014 (14) *3 months – 12 years*  - Osiak et al. 2018 (15) *2 weeks – 20 years post-HSCT*  - Wong er al. 2003 (16) |
| **Xerostomia not defined as patent reported oral dryness** |
| - Elad et al. 2011 (17) *unclear whether subjective or objective dryness*  - Baker et al. 2004, 2010, Majhail et al. 2007 (18-20) *question phrased as follows: have you ever been told by a doctor or health care professional that you have or have had abnormally dry mouth**  - Dyer et al. 2018, Smith et al. 2017 (21, 22) *question phrased as follows: Since your transplant, have you been diagnosed with the following?: dry mouth**  - Buchali et a. 2000 (23) *RTOG acute radiation morbidity scoring criteria (24)*  - Santos et al. 2004 (25) *Toxicity profile (WHO scale)*  - De Felice et al. 2016 (26) *Common Terminology Criteria for Adverse Events (CTCAE)(27)*  - Hayashi et al. 2014 (28) *CTCAE*  - Kim et al. 2017 (29) *CTCAE*  - Kitagawa et al. 2021(30) *CTCAE*  - Mark et al. 2013 (31) *CTCAE*  - Mark et al. 2017 (32) *CTCAE*  - Perrone et al. 2017 (33) *CTCAE*  - Ueda et al. 2012 (34) *CTCAE* |
| **Selection based on conditions developed after HSCT** |
| - Alborghetti et al. 2005 (35) *GvHD*  - Fassil et al. 2012 (36) *GvHD*  - Scaraficci et al. 2022 (37) *GvHD*  - Parkhideh et al. 2022 (38) *mucositis*  - Larsen et al. 2007 (39) *poor general health*  - Boland et al. 2013 (40) *symptomatic disease (MM) after HSCT* |
| **Results of children and adults combined** |
| - Boer et al. 2010 (41)  - Daikeler et al. 2013 (42)  - Hermann et al. 2005 (43)  - Seikh et al. 2021 (44) |

*****Studies were excluded after additional information was obtained from the authors, which confirmed that this studies did not meet the inclusion criteria

**Table S4, quality of included studies**

| **Study** | subgroups | Representativeness | Ascertainment bias | Misclassification bias | Examiner bias | Oral complication assessment validity | Estimate precision | **Total** |
| --- | --- | --- | --- | --- | --- | --- | --- | --- |
| Abasaeed 2018 |  | 0 | 1 | 1 | 1 | 1 | 0 | **4** |
| Andersson 2008, 2009, 2011 | total, autologous | 1 | 2 | 1 | 1 | 1 | 1 | **7** |
|  | MAC, RIC | 1 | 2 | 1 | 1 | 1 | 0 | **6** |
| Arduino 2022 |  | 1 | 0 | 0 | 0 | 0 | 0 | **1** |
| Bennett 2015 |  | 0 | 2 | 1 | 1 | 1 | 0 | **5** |
| Bergkvist 2015 |  | 1 | 0 | 1 | 1 | 1 | 1 | **5** |
| Cheon 2021 |  | 0 | 0 | 0 | 1 | 0 | 1 | **2** |
| Edman 2001 |  | 1 | 0 | 0 | 1 | 1 | 0 | **3** |
| Eriksson 2022 | Total, RIC | 1 | 1 | 1 | 1 | 1 | 1 | **6** |
|  | MAC | 1 | 1 | 1 | 1 | 1 | 0 | **5** |
| Hayden 2004 |  | 1 | 0 | 0 | 1 | 1 | 0 | **3** |
| Ferreira 2018 |  | 1 | 1 | 1 | 1 | 1 | 0 | **5** |
| Iestra 2002 |  | 1 | 2 | 0 | 1 | 1 | 1 | **6** |
| Jones 2013 |  | 0 | 1 | 1 | 1 | 1 | 1 | **5** |
| Kirsch 2014 | > 2 years total | 1 | 0 | 1 | 1 | 1 | 2 | **6** |
|  | >2 years MAC  >2 years RIC | 1 | 0 | 1 | 1 | 1 | 1 | **5** |
|  | 1 – 2 years | 1 | 0 | 1 | 1 | 1 | 0 | **4** |
| Kolke 2019 |  | 0 | 1 | 1 | 1 | 1 | 0 | **4** |
| Larsen 2003, 2004 |  | 1 | 2 | 1 | 1 | 1 | 0 | **6** |
| Lockhart 2005 |  | 0 | 0 | 1 | 1 | 1 | 0 | **3** |
| Naegele 2018 |  | 0 | 1 | 1 | 1 | 1 | 0 | **4** |
| Vellenga, van Agthoven 2001 |  | 1 | 1 | 1 | 1 | 1 | 1 | **6** |
| Watson 2004 |  | 1 | 0 | 0 | 1 | 1 | 1 | **4** |
| Warchala 2019 |  | 0 | 0 | 1 | 1 | 1 | 0 | **3** |
| Wood 2013 |  | 0 | 2 | 1 | 1 | 1 | 0 | **5** |
| Wysocka-Słowik 2021 | total | 0 | 1 | 0 | 1 | 0 | 1 | **3** |
|  | MAC, RIC | 0 | 1 | 0 | 1 | 0 | 0 | **2** |

**Table S5, phrasing of the used questionnaires**

**Table S5a, Questionnaires measuring severity of xerostomia**

| Questionnaire | Question | Response | Used by |
| --- | --- | --- | --- |
| EORTC QLQ-H&N35 | During the past week:  Have you had a dry mouth? | Not at all; A little; Quite a bit; Very much | Abasaeed 2018  Ferreira 2020 |
| EORTC QLQ-HDC-19^1^ | During the past week: Have you had a dry mouth? | 4-point response scale ranging from 1 (not at all) to 4 (very much) | Andersson 2008, 2009, 2011 |
| EORTC QLQ-Leu | During the Past MONTH, have you had problems with: mouth dryness? | Not at all; A little; Quite a bit; Very much | Hayden 2004 |
| PRO-CTCAE | In the last 7 days, what was the SEVERITY of your DRY MOUTH at its WORST? | None; Mild; Moderate; Severe; Very severe | Bennet 2015  Wood 2013 |
| PROVIVO |  |  | Kirsch 2014  Naegele 2018 |
| SFID-SCT | Have you experienced mouth dryness? | not at all; yes, a little; yes, quite a lot; yes, a lot  scale ranging from 1 ‘a little intense’ to 3 ‘very intense’ | Eriksson 2022  Larsen 2003, 2004 |
| MDASI-MM | Have you had a dry mouth? | 0 – 10 scale ranging from “not present” to “as bad as you can imagine” | Jones 2013 |
| VAS | How does your mouth feel most of the time? | 0: not dry at all, 100: dry as a desert | Lockhart 2005 |
| Authorial questionnaire |  | None; mild; moderate; severe | Wysocka-Słowik 2021 |

**Table S5b, Questionnaires measuring the distress caused by xerostomia**

| Quesionnaire | Question | Response | Used by |
| --- | --- | --- | --- |
| RSCL | Have you, during the past week, been bothered by: dry mouth? | Not at all; A little; Quite a bit; Very much | Vellenga 2001, van Agthoven 2001  Warchala 2019 |
| SIFD-SCT | If you experienced mouth dryness have you been distressed by it? | not at all; yes, a little; yes, quite a lot; yes, a lot | Eriksson 2022 |
|  |  | scale ranging from 0 ‘no distress till 3 ‘very distressing | Larsen 2003, 2004 |
| MSAS-SF | Did you have dry mouth? If yes, how much did it distress or bother you? | Not at all; a little bit; somewhat; quite a bit; very much | Kolke 2019 |
| PROVIVO | If dry mouth dryness occurred, how much did it BOTHER or BURDENED you? | None; Mild; Moderate; Severe; Very severe | Kirsch 2014  Naegele 2018 |

**Figure S1, meta-analysis: change in xerostomia (0 – 100) from baseline. Both allogeneic and autologous transplantations are included.**

**Figure S1a, Change in severity of xerostomia between baseline and week 1 post-HSCT**


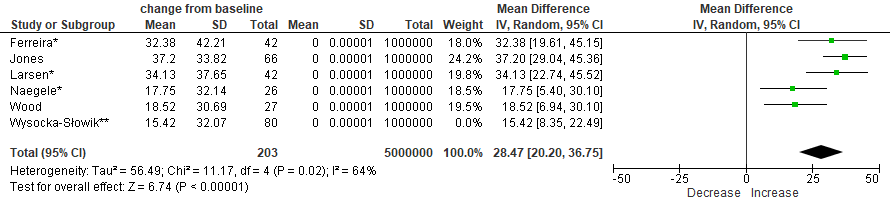

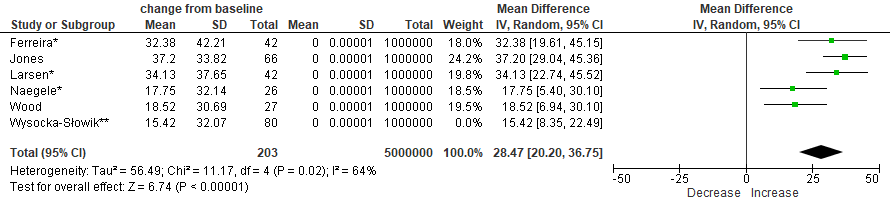


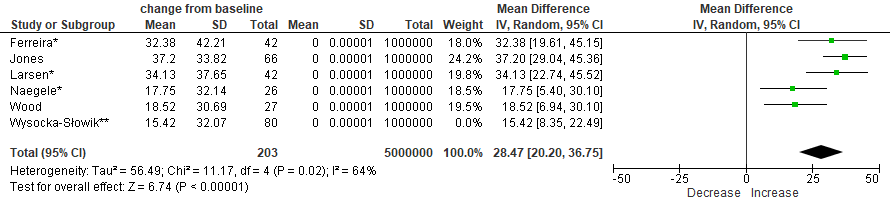


**SD’s for change score were imputed with the help of a correlation coefficient as suggested in the Cochrane Handbook for systematic Reviews of Interventions, chapter 6 (45)*

*** Results reported by Wysocka-Słowik received no weight in the meta-analysis because the quality rating resulted in 3 points, which was categorized as a high risk of bias*

**Figure S1b, Change in severity of xerostomia between baseline and 2 – 5 months post-HSCT**


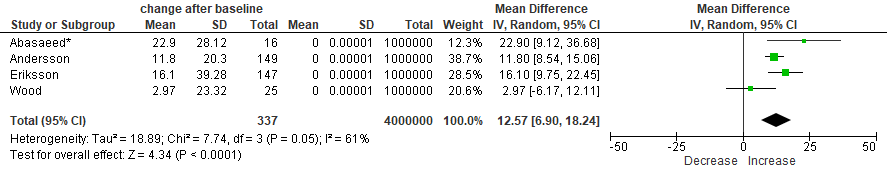

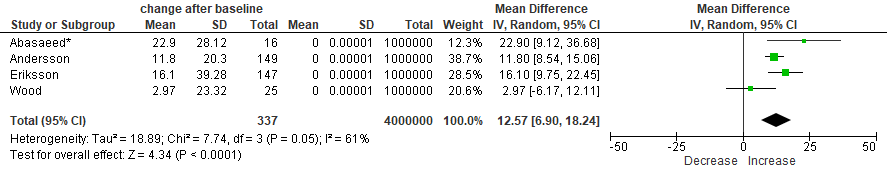

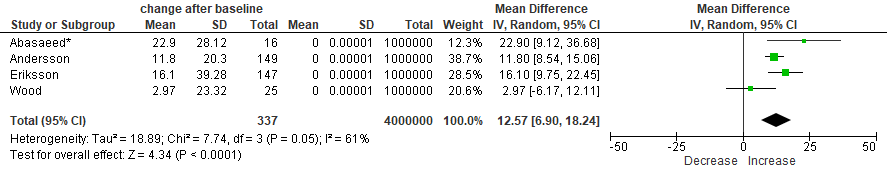


**SD for change score is imputed with the help of a correlation coefficient as suggested in the Cochrane Handbook for systematic Reviews of Interventions, chapter 6 (45)*


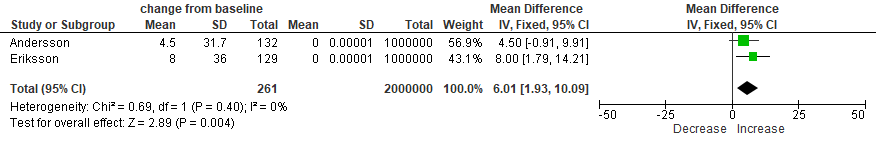
**Figure S1c, Change in severity of xerostomia between baseline and 1 – 2 years post-HSCT**


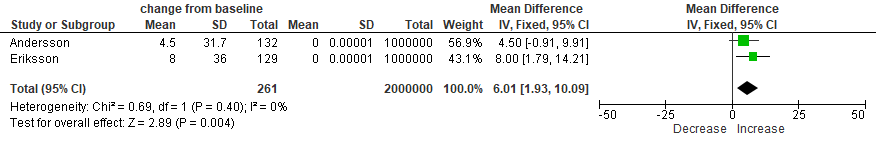

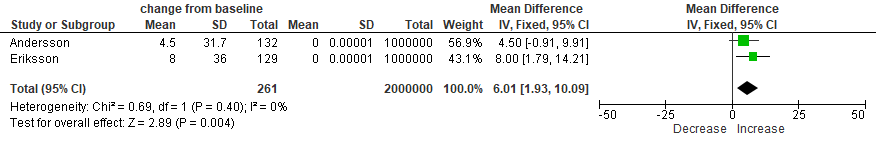


**Figure S2, meta-analysis:** **differences in severity of xerostomia (0 – 100) between allogeneic HSCT recipients receiving a myeloablative conditioning (MAC) in comparison to those receiving a reduced intensity conditioning (RIC) at different moments in time**

**Figure S2a, baseline**


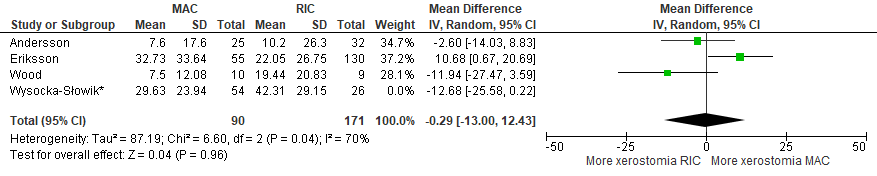


******* *Results reported by Wysocka-Słowik received no weight in the meta-analysis because the quality rating resulted in 3 points, which was categorized as a high risk of bias*

**Figure S2b, 2 – 5 months post-HSCT**


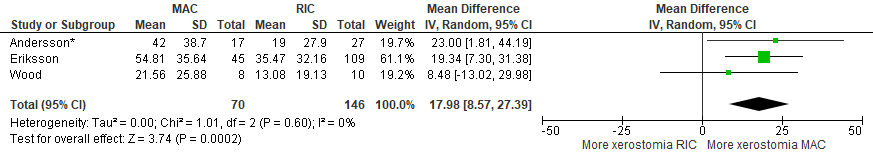


********SD’s are imputed* *with the help of a correlation coefficient as suggested in the Cochrane Handbook for systematic Reviews of Interventions, chapter 6 (45)*

**Figure S2c, 1 – 2 years post-HSCT**


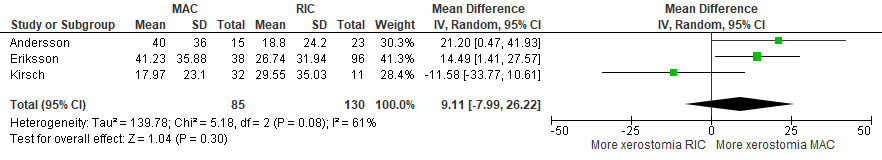


**Literature:**

1. Frowen J, Hughes R, Skeat J. The prevalence of patient-reported dysphagia and oral complications in cancer patients. Supportive Care in Cancer. 2020;28(3):1141-50.

2. Gerecitano J, Portlock C, Hamlin P, Moskowitz CH, Noy A, Straus D, et al. Phase I trial of weekly and twice-weekly bortezomib with rituximab, cyclophosphamide, and prednisone in relapsed or refractory non-hodgkin lymphoma. Clinical cancer research. 2011;17(8):2493‐501.

3. Hinkelmann JV, Possa LDO, de Oliveira CA, Faria BS, Hermsdorff HHM, Rosa CDOB. Food preferences and aversions of patients undergoing chemotherapy, radiotherapy and/or hematopoietic stem cell transplantation. Clinical Nutrition ESPEN. 2021;44:331-6.

4. Oerlemans S, Issa DE, van den Broek EC, Nijziel MR, Coebergh JW, Mols F, et al. Impact of therapy and disease-related symptoms on health-related quality of life in patients with follicular lymphoma: results of the population-based PHAROS-registry. Eur J Haematol. 2014;93(3):229-38.

5. Vadhan-Raj S, Trent J, Patel S, Zhou X, Johnson MM, Araujo D, et al. Single-dose palifermin prevents severe oral mucositis during multicycle chemotherapy in patients with cancer: a randomized trial. Ann Intern Med. 2010;153(6):358-67.

6. Bos-den Braber J, Potting CM, Bronkhorst EM, Huysmans MC, Blijlevens NM. Oral complaints and dental care of haematopoietic stem cell transplant patients: a qualitative survey of patients and their dentists. Support Care Cancer. 2015;23(1):13-9.

7. Brand HS, Bots CP, Raber-Durlacher JE. Xerostomia and chronic oral complications among patients treated with haematopoietic stem cell transplantation. Br Dent J. 2009;207(9):E17; discussion 428-9.

8. Caliskan K, Can G. Determining the symptoms and coping methods of patients at home after hematopoietic stem cell transplantation. Support Care Cancer. 2022.

9. Fantozzi PJ, Treister NS, Cutler CS, Villa A. Oral health in allogeneic hematopoietic stem cells transplantation survivors. Bone Marrow Transplantation. 2020;55(11):2211-4.

10. Gomes AO, Torres SR, Maiolino A, Dos Santos CW, Silva Junior A, Correa ME, et al. Early and late oral features of chronic graft-versus-host disease. Rev Bras Hematol Hemoter. 2014;36(1):43-9.

11. Hull KM, Kerridge I, Schifter M. Long-term oral complications of allogeneic haematopoietic SCT. Bone Marrow Transplant. 2012;47(2):265-70.

12. Kirsch M, Mitchell SA, Dobbels F, Stussi G, Basch E, Halter JP, et al. Linguistic and content validation of a German-language PRO-CTCAE-based patient-reported outcomes instrument to evaluate the late effect symptom experience after allogeneic hematopoietic stem cell transplantation. Eur J Oncol Nurs. 2015;19(1):66-74.

13. Meirelles CS, Diez-Garcia RW. Taste changes as a metaphor for biographical disruption: A qualitative study in patients undergoing haematopoietic stem cell transplantation. Clin Nutr ESPEN. 2018;27:127-33.

14. Oguz G, Akin S, Durna Z. Symptoms after hospital discharge following hematopoietic stem cell transplantation. Indian J Palliat Care. 2014;20(1):41-9.

15. Osiak M, Szubińska-Lelonkiewicz D, Wychowański P, Karakulska-Prystupiuk E, Jędrzejczak W, Wojtowicz A, et al. Frequency of Pathologic Changes in the Oral Cavity in Patients Subjected to Long-term Pharmacologic Immunosuppressive Therapy After Kidney, Liver, and Hematopoietic Cell Transplantation. Transplant Proc. 2018;50(7):2176-8.

16. Wong R, Giralt SA, Martin T, Couriel DR, Anagnostopoulos A, Hosing C, et al. Reduced-intensity conditioning for unrelated donor hematopoietic stem cell transplantation as treatment for myeloid malignancies in patients older than 55 years. Blood. 2003;102(8):3052-9.

17. Elad S, Luboshitz-Shon N, Cohen T, Wainchwaig E, Shapira MY, Resnick IB, et al. A randomized controlled trial of visible-light therapy for the prevention of oral mucositis. Oral oncology. 2011;47(2):125‐30.

18. Baker KS, Gurney JG, Ness KK, Bhatia R, Forman SJ, Francisco L, et al. Late effects in survivors of chronic myeloid leukemia treated with hematopoietic cell transplantation: results from the Bone Marrow Transplant Survivor Study. Blood. 2004;104(6):1898-906.

19. Baker KS, Ness KK, Weisdorf D, Francisco L, Sun CL, Forman S, et al. Late effects in survivors of acute leukemia treated with hematopoietic cell transplantation: A report from the Bone Marrow Transplant Survivor Study. Leukemia. 2010;24(12):2039-47.

20. Majhail NS, Ness KK, Burns LJ, Sun CL, Carter A, Francisco L, et al. Late effects in survivors of Hodgkin and non-Hodgkin lymphoma treated with autologous hematopoietic cell transplantation: a report from the bone marrow transplant survivor study. Biol Blood Marrow Transplant. 2007;13(10):1153-9.

21. Smith J, Poon C, Gilroy N, Kabir M, Brice L, Dyer G, et al. Nutritional issues and body weight in long-term survivors of allogeneic blood and marrow transplant (BMT) in NSW Australia. Support Care Cancer. 2017;25(1):137-44.

22. Dyer G, Brice L, Schifter M, Gilroy N, Kabir M, Hertzberg M, et al. Oral health and dental morbidity in long-term allogeneic blood and marrow transplant survivors in Australia. Aust Dent J. 2018.

23. Buchali A, Feyer P, Groll J, Massenkeil G, Arnold R, Budach V. Immediate toxicity during fractionated total body irradiation as conditioning for bone marrow transplantation. Radiother Oncol. 2000;54(2):157-62.

24. Cox JD, Stetz J, Pajak TF. Toxicity criteria of the Radiation Therapy Oncology Group (RTOG) and the European Organization for Research and Treatment of Cancer (EORTC). Int J Radiat Oncol Biol Phys. 1995;31(5):1341-6.

25. Santos ES, Goodman M, Byrnes JJ, Fernandez HF. Thalidomide effects in the post-transplantation setting in patients with multiple myeloma. Hematology. 2004;9(1):35-9.

26. De Felice F, Grapulin L, Musio D, Pomponi J, Di Felice C, Iori AP, et al. Treatment complications and long-term outcomes of total body irradiation in patients with acute lymphoblastic leukemia: A single institute experience. Anticancer Research. 2016;36(9):4859-64.

27. Cancer Therapy Evaluation Program, Common Terminology Criteria for Adverse Events, Version 4.0. [Internet]. 2010. Available from: <http://ctep.cancer.gov>.

28. Hayashi H, Kobayashi R, Suzuki A, Ishihara M, Nakamura N, Kitagawa J, et al. Polaprezinc prevents oral mucositis in patients treated with high-dose chemotherapy followed by hematopoietic stem cell transplantation. Anticancer Res. 2014;34(12):7271-7.

29. Kim JW, Kim MG, Lee HJ, Koh Y, Kwon JH, Kim I, et al. Topical Recombinant Human Epidermal Growth Factor for Oral Mucositis Induced by Intensive Chemotherapy with Hematopoietic Stem Cell Transplantation: final Analysis of a Randomized, Double-Blind, Placebo-Controlled, Phase 2 Trial. PloS one. 2017;12(1):e0168854.

30. Kitagawa J, Shimizu M, Tsurumi H, Kobayashi R, Ohata K, Kato-Hayashi H, et al. Polaprezinc for prevention of oral mucositis in patients receiving chemotherapy followed by hematopoietic stem cell transplantation: A multi-institutional randomized controlled trial. International Journal of Cancer. 2021;148(6):1462-9.

31. Mark TM, Reid W, Niesvizky R, Gergis U, Pearse R, Mayer S, et al. A phase 1 study of bendamustine and melphalan conditioning for autologous stem cell transplantation in multiple myeloma. Biology of Blood and Marrow Transplantation. 2013;19(5):831-7.

32. Mark TM, Forsberg P, Sherbenou D, Guarneri D, Rossi A, Pearse R, et al. A Phase I Trial of High-Dose Lenalidomide and Melphalan as Conditioning for Autologous Stem Cell Transplantation in Relapsed or Refractory Multiple Myeloma. Biology of Blood and Marrow Transplantation. 2017;23(6):930-7.

33. Perrone AC, Barbosa TR, da Silva FL, Perrone Í T, de Carvalho AF, Stephani R, et al. Supplementation with concentrated milk protein in patients undergoing hematopoietic stem cell transplantation. Nutrition. 2017;37:1-6.

34. Ueda T, Yokoyama K, Okamoto S, Ishizawa J, Matsuki E, Iino R, et al. Post-transplant consolidation therapy using thalidomide alone for the patients with multiple myeloma: A feasibility study in Japanese population. International Journal of Hematology. 2012;96(4):477-84.

35. Alborghetti MR, Corrêa ME, Adam RL, Metze K, Coracin FL, de Souza CA, et al. Late effects of chronic graft-vs.-host disease in minor salivary glands. J Oral Pathol Med. 2005;34(8):486-93.

36. Fassil H, Bassim CW, Mays J, Edwards D, Baird K, Steinberg SM, et al. Oral chronic graft-vs.-host disease characterization using the NIH scale. J Dent Res. 2012;91(7 Suppl):45s-51s.

37. Scaraficci AC, Fernandes PM, Abreu Alves F, Filho JS, Jaguar GC. Oral manifestations of graft-versus-host disease in patients submitted to allogeneic hematopoietic stem cell transplantation: the experience of a Brazilian Cancer Center. Support Care Cancer. 2022;30(1):567-73.

38. Parkhideh S, Zeraatkar M, Moradi O, Hajifathali A, Mehdizadeh M, Tavakoli-Ardakani M. Azithromycin oral suspension in prevention and management of oral mucositis in patients undergoing hematopoietic stem cell transplantation: a randomized controlled trial. Support Care Cancer. 2022;30(1):251-7.

39. Larsen J, Nordström G, Ljungman P, Gardulf A. Factors associated with poor general health after stem-cell transplantation. Support Care Cancer. 2007;15(7):849-57.

40. Boland E, Eiser C, Ezaydi Y, Greenfield DM, Ahmedzai SH, Snowden JA. Living with advanced but stable multiple myeloma: a study of the symptom burden and cumulative effects of disease and intensive (hematopoietic stem cell transplant-based) treatment on health-related quality of life. J Pain Symptom Manage. 2013;46(5):671-80.

41. Boer CC, Correa ME, Miranda EC, de Souza CA. Taste disorders and oral evaluation in patients undergoing allogeneic hematopoietic SCT. Bone Marrow Transplant. 2010;45(4):705-11.

42. Daikeler T, Mauramo M, Rovó A, Stern M, Halter J, Buser A, et al. Sicca symptoms and their impact on quality of life among very long-term survivors after hematopoietic SCT. Bone Marrow Transplant. 2013;48(7):988-93.

43. Hermann P, Berek Z, Kriván G, Márton K, Lengyel A. Incidence of oropharyngeal candidosis in stem cell transplant (SCT) patients. Acta Microbiol Immunol Hung. 2005;52(1):85-94.

44. Sheikh IN, Miller J, Shoberu B, Andersen CR, Wang J, Williams LA, et al. Using the MDASI-Adolescent for Early Symptom Identification and Mitigation of Symptom Impact on Daily Living in Adolescent and Young Adult Stem Cell Transplant Patients. Children (Basel). 2021;9(1).

45. Higgins J, Li T, Deeks J. Handbook for Systematic Reviews of Interventions version 6.3 Cochrane2022. Available from: [www.training.cochrane.org/handbook](file:///\\UMCFS012\THKdata$\ALG%20PCT\Orastem\MarjoleinsData\systematic%20review\www.training.cochrane.org\handbook).
